# Pan-genomic perspective on the evolution of the *Staphylococcus aureus* USA300 epidemic

**DOI:** 10.1099/mgen.0.000058

**Published:** 2016-05-31

**Authors:** Dorota M. Jamrozy, Simon R. Harris, Naglaa Mohamed, Sharon J. Peacock, Charles Y. Tan, Julian Parkhill, Annaliesa S. Anderson, Matthew T. G. Holden

**Affiliations:** ^1^​ The Wellcome Trust Sanger Institute, Cambridge CB10 1SA, UK; ^2^​ Pfizer Vaccine Research and Development, Pearl River, New York, USA; ^3^​ London School of Hygiene and Tropical Medicine, London WC1E 7HT, UK; ^4^​ School of Medicine, University of St Andrews, St Andrews, Fife KY16 9TF, UK

**Keywords:** MRSA, USA300, Evolution

## Abstract

*Staphylococcus aureus* USA300 represents the dominant community-associated methicillin-resistant *S. aureus* lineage in the USA, where it is a major cause of skin and soft tissue infections. Previous comparative genomic studies have described the population structure and evolution of USA300 based on geographically restricted isolate collections. Here, we investigated the USA300 population by sequencing genomes of a geographically distributed panel of 191 clinical *S. aureus *isolates belonging to clonal complex 8 (CC8), derived from the Tigecycline Evaluation and Surveillance Trial program. Isolates were collected at 12 healthcare centres across nine USA states in 2004, 2009 or 2010. Reconstruction of evolutionary relationships revealed that CC8 was dominated by USA300 isolates (154/191, 81 %), which were heterogeneous and demonstrated limited phylogeographic clustering. Analysis of the USA300 core genomes revealed an increase in median pairwise SNP distance from 62 to 98 between 2004 and 2010, with a stable pattern of above average d*N*/d*S* ratios. The phylogeny of the USA300 population indicated that early diversification events led to the formation of nested clades, which arose through cumulative acquisition of predominantly non-synonymous SNPs in various coding sequences. The accessory genome of USA300 was largely homogenous and consisted of elements previously associated with this lineage. We observed an emergence of SCC*mec* negative and ACME negative USA300 isolates amongst more recent samples, and an increase in the prevalence of ϕSa5 prophage. Together, the analysed *S. aureus* USA300 collection revealed an evolving pan-genome through increased core genome heterogeneity and temporal variation in the frequency of certain accessory elements.

## Data Summary

1. Whole-genome sequence reads and assemblies of *Staphylococcus aureus* isolates described in this study have been deposited in the European Nucleotide Archive. The accession numbers together with related metadata are provided in Table S1 (available in the online Supplementary Material).

## Impact Statement

The expansion of *Staphylococcus aureus* USA300 and its dominance as a community-associated MRSA clone within the USA has prompted interest in the genomics of this pathogen. The resolving power of whole-genome sequencing has already shed light on the nature of USA300 transmission within defined communities. However, a question of the broader population structure remains. Here, we investigated whole-genome sequences of epidemiologically diverse CC8 isolates, inclusive of 154 USA300 samples. Based on this collection we investigated the wider population structure of USA300 and other CC8 isolates and assessed temporal variation in core and accessory genomes. We report that the USA300 population has become increasingly heterogeneous between 2004 and 2010 at the core genome level, accompanied by subtle changes in the accessory genome involving loss of ACME and SCC*mec* elements and an increase in the prevalence of ϕSa5 prophage. These findings demonstrate that successful lineages of MRSA are not genetically static and continue to evolve, which may involve loss of molecular markers previously considered to define the lineage. While further surveillance of USA300 genomes is required to confirm wider SCC*mec* and ACME loss, such genomic changes might impact on the future epidemiology of this important *S. aureus* clone.

## Introduction

*Staphylococcus aureus* is a major cause of human disease worldwide. One of the most successful lineages of this bacterial species is clonal complex 8 (CC8), from which a number of major methicillin-resistant *S. aureus* (MRSA) clones have emerged (Deurenberg & Stobberingh, 2008). This includes several pandemic MRSA clones associated with causing healthcare-associated (HA) infections as well as community-associated (CA) infections (Monecke* et al.*, 2011). HA-MRSA CC8 clones include historic Archaic and Iberian clones as well as the more contemporaneous USA500 clone, whereas the current USA300 epidemic clone has emerged as the dominant cause of CA infections in the USA.

The first reports of USA300 date back to the early 2000s, when it was identified in a number of localised outbreaks of CA skin and soft-tissue infections (SSTI) (Centers for Disease Control and Prevention (CDC), 2001, 2003, McDougal* et al.*, 2003; Tenover* et al.*, 2006). Additional reports followed which demonstrated the widespread dissemination of USA300 across the USA (Begier* et al.*, 2004; Kazakova* et al.*, 2005; Miller* et al.*, 2005). Other than SSTI, USA300 has also been reported as a causative agent of CA pneumonia, sepsis, infective endocarditis and osteomyelitis (Francis* et al.*, 2005; Gonzalez* et al.*, 2005; Haque* et al.*, 2007; Seybold *et al.*, 2007). In addition to being responsible for a rising incidence of CA infections, USA300 has also been increasingly reported in association with nasal colonisation (Freitas* et al.*, 2010; Tenover *et al*., 2008) and HA infections (Gonzalez *et al.*, 2006; Patel* et al.*, 2007; Seybold* et al.*, 2006). In the healthcare setting, USA300 was reported to emerge alongside established HA-MRSA clones (Chua* et al.*, 2008; Limbago *et al.*, 2009; Pasquale* et al.*, 2013; Tattevin* et al.*, 2009) or to replace them (Carey* et al.*, 2010; Liu* et al.*, 2008).

The striking epidemiology of USA300 within the USA has prompted a number of genomic studies aimed at the identification of genetic features that might have promoted its transmission, host colonisation and disease pathogenesis. Early whole-genome sequence analysis of a single USA300 strain revealed that the accessory genome included a Staphylococcal Cassette Chromosome *mec* (SCC*mec*) type IVa element, an arginine catabolic mobile element (ACME) type I, a *S. aureus* pathogenicity island 5 (SaPI5) and a Panton-Valentine leukocidin-carrying ϕSa2 prophage (Diep* et al.*, 2006). These four mobile genetic elements (MGEs) represent common molecular markers of USA300, although analysis of a wider population demonstrated that their carriage is not ubiquitous (Uhlemann* et al.*, 2014). Isolates belonging to USA300 also frequently carry two plasmids: a 27  kb plasmid carrying genes that confer resistance to penicillin (*blaZ*), erythromycin (*msrA* and *mphBM*) bacitracin (*bcrA*), kanamycin (*aphA-3*) and streptothricin (*sat*), and a 3.1 kb cryptic plasmid (Kennedy* et al.*, 2010). The contribution of MGEs to the success of USA300 has been a subject of debate and generation of the hypothesis that the accessory genome has promoted the enhanced transmission of USA300 (Li* et al.*, 2009). In particular, the ACME element has been suggested to promote the survival of USA300 on human skin and persistence within cutaneous abscesses (Thurlow* et al.*, 2013). In contrast, the pathogenesis of USA300 may have been mediated by subtle genetic changes within the core genome (Highlander* et al.*, 2007) together with modulated expression of core virulence determinants such as α-toxin, phenol-soluble modulins (PSMs) and an accessory gene regulator (*agr*) (Li *et al*., 2009). Core genome single nucleotide polymorphisms (SNPs) might also result in significantly different virulence phenotypes between USA300 isolates, associated with variable exoprotein expression (Kennedy* et al.*, 2008).

Recently, the genomics of USA300 have been studied on a wider scale, through whole-genome sequencing of larger collections derived from specific geographic regions (Alam* et al.*, 2015; Uhlemann *et al*., 2014). This led to significant epidemiological findings such as the identification of households as long-term reservoirs of USA300. Comparative genomic analysis has also allowed the diversification of the USA300 lineage to be studied, including evolutionary events surrounding the emergence of the USA300 clade as well as micro-evolution within the population (Uhlemann *et al*., 2014). This exemplifies the power of whole-genome sequencing for comprehensive analysis of bacterial pathogens, as it allows an in-depth interrogation of phylogenetic relationships, identification of genetic features associated with virulence and antimicrobial resistance, and the reconstruction of significant evolutionary events (Harris* et al.*, 2010; Holden* et al.*, 2013). While previous studies describing the comparative genomics of USA300 focused on geographically restricted isolate collections, here we describe whole-genome sequence analysis of *S. aureus* CC8 derived from 12 USA healthcare centres located across nine states. The isolates, collected in 2004, 2009 or 2010, were derived from the multi-centre Tigecycline Evaluation and Surveillance Trial (T.E.S.T) study and represent consecutive clinical isolates (methicillin-susceptible and methicillin -resistant) from both the community and hospital settings. As USA300 has become widespread across the USA, an analysis of a collection from geographically distinct locations has allowed us to study a broader population of epidemiologically unrelated USA300 isolates as well as their microevolution over time. For this we interrogated the phylogeny of the CC8 lineage, with particular focus on the USA300 clade, followed by analysis of temporal variation within core and accessory genomes as well as investigation of more subtle polymorphisms that define the phylogenetic structure of the USA300 population.

## Methods

### Bacterial isolates.

Isolates analysed here derived from the Tigecycline Evaluation and Surveillance Trial (T.E.S.T.), a global multi-centre surveillance study. The trial was initiated in 2004, and since then sites have been added and removed. For the purpose of conducting a longitudinal assessment of *S. aureus* isolates associated with disease in the USA, we prospectively selected USA sites that were participating between 2004 and 2010 (five sites). A further seven sites active at the time of study were included. A total of 516 *S. aureus* isolates derived from 2004, 2009 and 2010 were collected and analysed. All isolates belonging to CC8 (*n* = 191), as determined by multi-locus sequence typing, were selected for this work.

**Fig. 1.**
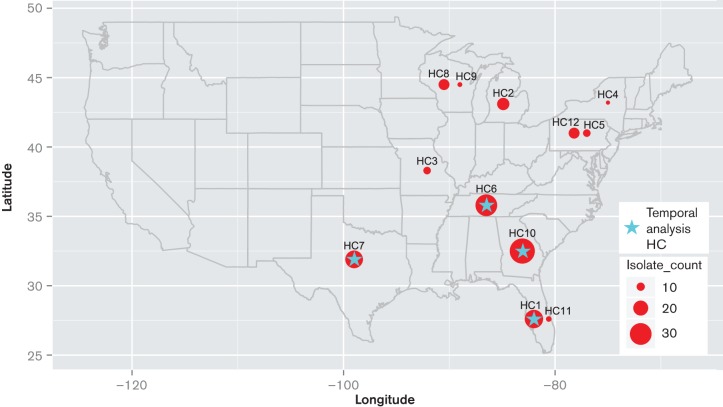
Geographic distribution of healthcare centres (HC) that collected isolates analysed in this study. The geographic location on the map represents state coordinates, where the corresponding HC was based. Plots are scaled to represent total number of isolates from corresponding site. HCs that provided isolates used in temporal analyses are marked with a star.

In line with T.E.S.T requirements, the participating sites collected consecutive isolates from patients with a documented infection. Only one isolate per patient was permitted and all body sites were considered an acceptable clinical source. Epidemiological details of the 191 *S. aureus* CC8 isolates analysed here are presented in Table S1 (available in the online Supplementary Material). Briefly, isolates were derived from the 12 T.E.S.T. sites that were located across nine states of the USA (Fig. 1), with 26 % (50/191) collected in 2004, 31 % (58/191) in 2009 and 43 % (83/191) in 2010. For the purpose of this work the collecting sites were called healthcare centre 1 – 12 (HC1 – HC12). The collection consisted of 46 MSSA and 145 MRSA isolates. Isolates were collected from diverse clinical sources, categorised in this work to represent four main infection types defined as wound, invasive, respiratory, or other. Isolates were collected from patients aged 0 to 95 in various clinical settings, with samples broadly defined in this study as derived from either inpatient or outpatient.

### Whole-genome sequencing.

Genomic DNA was isolated using the Qiagen QIAcube system according to manufacturer’s protocol. Tagged DNA libraries were created using a method adapted from a standard Illumina Indexing protocol, as described previously (Hsu* et al.*, 2015). Whole-genome sequencing was performed on the Illumina HiSeq 2000 platform with 100  bp paired-end reads. The Illumina sequence data has been submitted to the European Nucleotide Archive (ENA) and the accession numbers are provided in Table S1.

### Whole-genome sequence data analysis.

Single nucleotide polymorphisms (SNPs) were detected by mapping the paired-end reads against the *S. aureus* USA300_FPR3757 reference genome (GenBank accession NC_007793), using SMALT version 0.5.8. (http://www.sanger.ac.uk/science/tools/smalt-0). The generated whole-genome sequence alignment was curated to exclude accessory regions (mobile genetic elements identified for USA300_FPR3757 reference genome) and variable sites associated with recombination, with the latter detected using Gubbins (Croucher* et al.*, 2014). Based on the curated core genome SNP alignment a maximum-likelihood phylogenetic tree was reconstructed using RAxML version 7.8.6 (Stamatakis, 2014) with the generalised time reversible (GTR) model with GAMMA method of correction for among site rate variation and 100 bootstrap replications (Harris *et al*., 2010). *De novo* assembly of whole-genome sequences was performed using Velvet (Zerbino & Birney, 2008) with Velvet Optimiser (http://bioinformatics.net.au/software.velvetoptimiser.shtml) and kmers ranging between 66 % and 90 % of the read-length. Contigs of less than 300 bp were removed. The draft assemblies have been submitted to the ENA with accession numbers provided in Table S1. The assembled contigs were annotated using Prokka (Seemann, 2014). The accessory genome was defined as described previously (Harris *et al*., 2010).

### Isolate genotyping.

Multi-locus sequence typing was performed on assembled genomes using an in-house developed pipeline based on a previously described typing scheme (Enright* et al.*, 2000). Isolates were also *spa* genotyped by extracting the *spa* gene variable X region from assembled genomes using previously described primers (Kahl* et al.*, 2005), and determining the *spa* type with the spaTyper tool (http://spatyper.fortinbras.us). MLST and *spa* genotypes of all isolates are presented in Table S1. In addition, *in silico* PCR was used to confirm carriage of prophages (Goerke* et al.*, 2009) and to determine the SCC*mec* type amongst *mecA-*positive samples (Milheiriço *et al*., 2007a, b).

### Statistical analyses.

To test the statistical significance of the temporal variation in the prevalence of USA300 isolates as well as selected mobile genetic elements, the likelihood ratio test was applied. The strength of phylogenetic signal of epidemiological trait was measured using phylo.d() function in R package caper based on 1000 permutations. The function computes a *D* value, which provides a measure of trait dispersion for categorical characters (Fritz & Purvis, 2010). A negative *D* statistic indicates that a trait is phylogenetically clustered whereas a positive *D* statistic indicates that a trait is over dispersed across the phylogenetic tree.

## Results and Discussion

### CC8 population structure overview and temporal variation

**Fig. 2.**
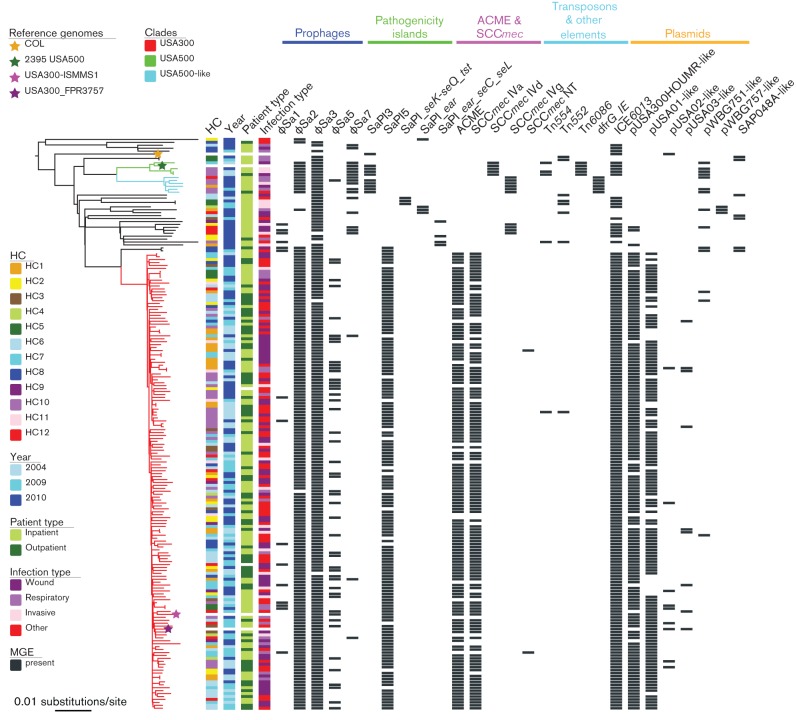
Midpoint-rooted phylogenetic tree of the 191 analysed *S. aureus* CC8 isolates. Four reference genomes were included (corresponding nodes marked with a star). Branches of USA300, USA500 and USA500-like clades have been highlighted. The tree has been annotated to show the association between phylogenetic distribution and healthcare centre (HC) of origin (excluding reference genomes), year of collection (excluding reference genomes), patient type (excluding reference genomes), infection type (excluding reference genomes) and carriage of mobile genetic elements (MGE). The latter shows the distribution of identified prophages, pathogenicity islands (where novel sequence was identified, name has been assigned based on virulence gene content), transposons and other elements such as ICE*6013* and *dfrG* insertion element, and plasmids (name assigned based on close similarity to previously identified plasmid). Further details on variable carriage of virulence genes within ϕSa2 and ϕSa3 prophages, as well as variable distribution of antimicrobial resistance genes within pUSA300HOUMR-like and pUSA03-like plasmids have been provided in Fig. S1. The tree was annotated using EvolView (Zhang* et al.*, 2012).

Whole-genome sequences of 191 clinical *S. aureus* isolates belonging to CC8 were analysed, the majority of which belonged to ST8 (179/191, 94 %; Table S1). A total of ten single-locus variants (SLV) of ST8 were identified, which included six distinct novel STs. The majority of isolates belonged to *spa* type t008 (146/191, 76 %; Table S1). Alignment of the core genomes (after excluding accessory regions and regions of predicted recombination, resulting in a core genome length of 2 592 236 bp) of all CC8 isolates revealed a total of 10 651 SNP sites. To identify different sub-lineages, a phylogenetic tree of all CC8 samples was reconstructed with the addition of USA300, USA500 and COL reference genomes (Fig. 2). The tree revealed that the majority of isolates belonged to a single dominant clade (154/191, 81 %), which contained the two USA300 reference strains (USA300_FPR3757 and USA300-ISMMS1) and thus was identified to represent the USA300 genotype. The population also contained four isolates that clustered with the USA500 reference strain (2395 USA500) and, as such, represented the USA500 clone. Six further isolates were closely related to the USA500 clade but descended from a distinct node and were labelled here USA500-like. The majority of these USA500 and USA500-like isolates (7/10) originated from a single healthcare centre (HC10, state of Georgia). The reconstructed phylogeny and detection of USA300 and USA500 genotypes in the context of a diverse CC8 population revealed that the two are only distantly related, in contrast to a previous suggestion that USA500 represents a progenitor of the USA300 clone (Li *et al*., 2009). A number of non-clonal MSSA CC8 isolates were more closely related to the USA300 clade than the USA500 sub-lineage suggesting that the two MRSA clones arose independently. The structure of CC8 phylogeny presented here is in line with a recently published overview of evolutionary relationships between various CC8 genomes (Boyle-Vavra *et al.*, 2015).

Isolates belonging to the USA300 clade were detected amongst samples from all healthcare centres. Some genotypic heterogeneity was observed within the USA300 clade, which contained six SLVs of ST8. We also observed a level of *spa* type diversity amongst USA300 isolates. While the majority belonged to *spa* type t008 (132/154, 86 %), 10 other *spa* types were identified, including t024 (8/154, 5 %) and t681 (3/154, 2 %). The detected genotype diversity of USA300 isolates based on multi- and single-locus genotyping underscores the benefit of applying core genome sequence phylogeny for comprehensive identification of clinically significant sub-lineages such as *S. aureus* USA300.

Focusing on the USA300 clade only, we investigated the strength of phylogenetic signal in the distribution of isolates based on the healthcare centre of origin, year of isolation, patient type and infection type. For this we calculated the *D* statistic, which provides a measure of trait dispersion. A random distribution of isolates based on geographic origin was observed for the majority of healthcare centres (*D* range 0.17–1.22), with exception of HC1 (*D *= −0.68). Phylogenetic clustering was also observed for isolates collected in 2004 (*D *= −0.08) with random distribution of isolates from 2009 and 2010 (*D *= 0.86 and *D *= 0.85, respectively). No phylogenetic signal could be observed based on patient type (outpatient,* D *= 0.73; inpatient,* D *= 0.91) or infection type (wound, *D *= 0.48; respiratory, *D *= 0.67; invasive, *D *= 1.1).

We next investigated whether the CC8 population structure and the distribution of USA300 genotype varied at different sampling time points. For this, isolates collected in 2004 together with 2009 and 2010 collections derived from matched healthcare centres were used (Fig. 1). We found that USA300 was the dominant clone in all years. No significant variation in the distribution of USA300 was observed between 2004 (46/50, 92 %) and 2009 (23/24, 96 %) collections (*p* = 0.52). However, the 2010 collection (29/40, 73 %) revealed a significantly lower frequency of USA300 genotype in comparison with both 2004 (*p *< 0.05) and 2009 (*p* < 0.05). This suggests that the significant drop in the prevalence of USA300 observed for 2010 was likely specific to that year rather than a time trend. Outside of the USA300 clade, the USA500-like clone was observed only amongst more recent samples with a single isolate identified in 2009 (1/24, 4 %) and a comparatively higher prevalence in 2010 (4/40, 10 %).

### Accessory genome of CC8

The genomes of 191 *S. aureus* CC8 isolates were analysed for the distribution of variable regions, followed by annotation to define the accessory component. Overall, a diverse mobile genetic repertoire was identified across the lineage, although isolates belonging to USA300, USA500 and USA500-like clones revealed the presence of clade-associated MGEs (Fig. 2).

In total, five different prophage types were identified. The most common was ϕSa3, found in 96 % of isolates, although variation in carriage of prophage-encoded virulence factors was observed (Fig. S1). Nearly all ϕSa3-positive USA300 isolates carried the immune evasion cluster (IEC) composed of *scn, chp* and *sak* genes, which was also detected amongst some non-USA300 isolates. The second most common prophage, due to the dominance of USA300 within this population, was ϕSa2. All but one USA300 isolate carried the Panton-Valentine leukocidin-positive ϕSa2USA300, which was also detected in three non-USA300 isolates. Three further prophages were identified within the analysed CC8 population: ϕSa1, ϕSa5 and ϕSa7. All three were detected at considerably lower frequency than ϕSa3 or ϕSa2. Prophage ϕSa1 was detected sporadically but across the entire CC8 population, whereas ϕSa7 was prevalent mostly amongst non-USA300 MRSA isolates and found in all USA500 and the majority of USA500-like (5/6) samples. In contrast, prophage ϕSa5 was identified predominantly within the USA300 clade (47/154, 30 %), and was randomly distributed (*D *= 1.66) across the USA300 phylogeny suggesting multiple acquisition events rather than prevalence due to clonal propagation of ϕSa5-positive isolates. No putative virulence genes were identified within the sequence of any of the three prophages. We investigated whether acquisition of these prophages might affect the bacterial host thorough gene inactivation at the insertion site. Analysis of the chromosomal environment surrounding the site of prophage insertion revealed that ϕSa1 and ϕSa7 inserted within intergenic locations downstream of the iron-sulphur cluster assembly protein gene *sufB* (between coding sequences corresponding to USA300_FPR3757 SAUSA300_0822 and SAUSA300_0823), and the heme transport protein gene *isdB* (between coding sequences corresponding to USA300_FPR3757 SAUSA300_1027 and SAUSA300_1028), respectively. In contrast, prophage ϕSa5 were found inserted within a coding sequence responsible for a putative metal-binding protein with iron-sulphur oxidoreductase domain (SAUSA300_1858), which would likely interfere with its expression.

Other accessory virulence genes amongst analysed CC8 isolates were associated with various pathogenicity islands, with SaPI5 identified in majority of USA300 isolates (139/154, 90 %). Nearly all USA500 (3/4) and USA500-like (5/6) isolates carried the SaPI3, which similarly to SaPI5, encodes the *seK* and *seQ* enterotoxin genes.

The majority of USA300 isolates carried ACME (137/154, 89 %) and SCC*mec* type IVa elements (130/154, 84 %), which were not found in any other isolates. Where the elements were absent this was predicted to have resulted from a deletion event, with corresponding isolates dispersed across the phylogenetic tree. In line with a previous report (Uhlemann *et al*., 2014), an association between the absence of SCC*mec* and ACME was observed, with majority of ACME-negative USA300 isolates also lacking the SCC*mec* (13/17, 76 %). This is not surprising considering the proximal location of these elements in the USA300 chromosome. Nearly all SCC*mec-*positive USA300 isolates carried a complete type IVa element although two isolates carried unusual recombinant variants of SCC*mec* IVa, which arose through putative recombination of SCC*mec *with ACME or another SCC*mec* element (Fig. S2). We also identified one isolate carrying a remnant SCC element lacking the *mecA* gene that exhibited similarity to SCC*pbp4* from *Staphylococcus epidermidis *ATCC 12228. Finally, non-USA300* mecA*-positive isolates carried either SCC*mec *IVd or SCC*mec* IVg. The carriage of SCC*mec *correlated with the methicillin susceptibility phenotype (Table S1) except for a single SCC*mec-*positive isolate, which was identified as MSSA (PFESA2067). Further investigation revealed the presence of a single base insertion in *mecA* gene resulting in a frame-shift mutation and premature stop codon, which would likely lead to a loss of *mecA* expression.

The analysed CC8 population revealed a high prevalence of an accessory region recognised as integrative conjugative element 6013 (ICE*6013*) (Smyth & Robinson, 2009). The element was detected in all USA300 and USA500 isolates but was absent in all USA500-like samples. The ICE*6013* does not appear to carry any specific virulence or resistance genes and lacks insertion specificity (Smyth & Robinson, 2009), integrating at a number of sites amongst the analysed CC8 isolates, including both inter- and intragenic positions (Fig. S3). In the USA300 isolates however, ICE*6013* was only found inserted within a putative membrane protein gene (SAUSA300_0606), and thus an insertional inactivation of this particular coding sequence might provide selective advantage to USA300. Interestingly, this particular ICE*6013* insertion site differs from the USA300_FPR3757 reference genome (Diep *et al*., 2006), which also contains this element, but is identical to ICE*6013* location within the USA300-ISMMS1 genome (Altman* et al.*, 2014).

There was only a sporadic carriage of resistance-associated transposon elements such as *tetM*-positive Tn*6086, ermA-* and *spc*-positive Tn*554*, and *bla* operon-carrying Tn*552*. All USA500-like isolates carried a 3.3 kb insertion element containing the trimethoprim resistance gene *dfrG*, not found elsewhere in this collection. Amongst USA300 isolates, the antimicrobial resistance genes were carried predominantly on plasmids, with the majority of samples (146/154, 95 %) carrying a large plasmid resembling a previously reported 27 kb pUSA300HOUMR (Highlander *et al*., 2007). The plasmid contains genes that confer resistance to various antimicrobials including penicillin (*blaZ*), kanamycin (*aphA-3*), streptothricin (*sat*), bacitracin (*bcrA)* and erythromycin (*msrA, mphBM*), and homologous plasmids have been previously reported in other USA300 isolates (Kennedy *et al*., 2010; Uhlemann *et al*., 2014). However, amongst the 146 USA300 isolates carrying the pUSA300HOUMR-like plasmid, 12 carried a variant that lacked some of the resistance genes described above, with at least five different variants detected (Fig. S1). The pUSA300HOUMR-like plasmid was not confined to the USA300 clade within this collection, and was detected in several other isolates. A few USA300 isolates (8/154, 5 %) carried a *circa* 34 kb plasmid, which partly resembled the 37 kb pUSA03 that contains genes conferring resistance to erythromycin (*ermC*) and mupirocin (*ileS-2*) (Diep *et al*., 2006). At least three variants of this pUSA03-like plasmid were observed (Fig. S1). All lacked the *ermC* gene, and only some carried the *ileS-2* resistance determinant. In addition some of the *ileS-2-*positive variants also carried the kanamycin resistance gene *addD*. Also detected amongst USA300 isolates (3/154, 2 %) was a 2.5  kb *ermC-*positive pWBG751-like plasmid and a small tetracycline resistance (*tetK*) plasmid (8/154, 5 %), resembling the 4.4 kb pUSA02 (Diep *et al*., 2006).

### Temporal variation in the accessory genome

We further analysed whether the variable distribution of selected MGEs was associated with the year of sampling. For this, we compared the distribution of SCC*mec*, ACME and ϕSa5 amongst USA300 isolates collected in 2004 with the 2009 and 2010 collections from matched healthcare centres (Table 1). All USA300 isolates carried SCC*mec* in 2004, with a single negative isolate in 2009. The frequency of SCC*mec* carriage was lowest amongst 2010 isolates, which represented a significant drop in comparison with both 2004 (*p* < 0.05) and 2009 (*p *< 0.05) groups. Emergence of SCC*mec-*negative USA300 isolates resulting from a sporadic loss of the element has been previously reported, however at lower frequency than observed here (Uhlemann *et al*., 2014). As such the reduced frequency of SCC*mec* amongst USA300 isolates from 2010 might be specific to that year. Nevertheless a reported shift in antimicrobial prescription practices in the US between 2004 and 2008, away from *β*-lactams in favour of MRSA-active drugs when treating SSTI might have influenced the epidemiology of CA-MRSA infections (Talan *et al*., 2011). Lower frequency of beta-lactam usage could result in a lower selective pressure to maintain SCC*mec* leading to gradual loss of this element. While USA300 has been recognised predominantly as an MRSA lineage, infections caused by MSSA USA300 have also been reported suggesting that transmission of USA300 may not be entirely dependent on SCC*mec* carriage (McCaskill* et al.*, 2007; Talan *et al*., 2011). In support of the hypothesis that a shift in epidemiology of MRSA might have occurred around 2010, we also observed an increase in the prevalence of non-USA300 SCC*mec-*negative isolates at this sampling time point.

**Table 1.** Temporal variation in the distribution of selected mobile genetic elements among USA300 isolates. Based on isolates derived from healthcare centres selected for temporal analysis (HC1, HC6, HC7, HC10)

	2004				2009				2010			
	N^*^	SCC*mec*	ACME	ΦSa5	N^*^	SCC*mec*	ACME	ΦSa5	N^*^	SCC*mec*	ACME	ΦSa5
HC1	14	14	14	2	na	na	na	na	8	6	7	5
HC6	6	6	6	1	12	12	12	4	8	7	7	3
HC7	8	8	8	3	10	9	8	2	5	2	4	3
HC10	18	18	18	2	1	1	1	0	8	6	6	3
Total	46	46	46	8	23	22	21	6	29	21	24	14
*p*^†^						0.19	<0.05	0.7				
*p*^‡^										<0.05	<0.05	<0.05
*p*^§^										<0.05	0.39	0.07
												

* Total number of USA300 isolates identified for corresponding centre and year.

† *p* value after controlling for each HC, for the distribution of corresponding MGE among USA300 isolates in 2004 and 2009.

‡ *p* value after controlling for each HC, for the distribution of corresponding MGE among USA300 isolates in 2004 and 2010.

§ *p* value after controlling for each HC, for the distribution of corresponding MGE among USA300 isolates in 2009 and 2010.

As described above, carriage of ACME was associated with the distribution of SCC*mec* amongst USA300 isolates. Therefore in line with SCC*mec* prevalence, loss of ACME was only observed in 2009 and 2010 samples, both groups showing a significantly lower number of ACME-positive USA300 isolates when contrasted with the 2004 group (*p* < 0.05). Although the prevalence of ACME-negative USA300 isolates was more notable in 2010, the difference between 2009 and 2010 was not significant (*p* = 0.39).

The sample size of our collection was relatively low and thus the observed temporal variation in the distribution of SCC*mec* and ACME among USA300 isolates, and the apparent loss of these elements in more recent samples, would require further surveillance. However, the occurrence of SCC*mec-*negative and ACME-negative USA300 isolates was not limited to a particular healthcare centre (Table 1). Furthermore, the absence of phylogenetic association between USA300 isolates lacking either SCC*mec* or ACME suggests that forces driving the loss of these important MGEs are acting upon the wider USA300 population.

In addition to changes in prevalence of SCC*mec* and ACME, we observed a trend of increasing prophage ϕSa5 frequency, although only the difference between 2004 and 2010 isolates was statistically significant (*p* < 0.05). The overall frequency of ϕSa5 carriage by USA300 isolates collected in 2009 and 2010 was higher than the prevalence reported for New York City isolates (16 %) (Uhlemann *et al*., 2014). However, the presence of ϕSa5 prophage within the latter collection was associated with a particular USA300 sub-clade, suggesting that carriage of this prophage might contribute to expansion of more successful sub-populations (Uhlemann *et al*., 2014).

The USA300 population analysed in this study demonstrated a stable temporal prevalence of antimicrobial resistance-associated MGEs. All isolates collected in 2004 carried the multiple drug resistance pUSA300HOUMR-like plasmid, which was also found in the majority of 2009 (20/23, 87 %) and 2010 isolates (28/29, 97 %) from matched healthcare centres. A dip in pUSA300HOUMR-like plasmid prevalence amongst 2009 isolates was specific to a single centre and thus unlikely to represent a wider trend. It has been previously observed that carriage of aminoglycoside- and bacitracin-resistance genes associated with the pUSA300HOUMR-like plasmid might mediate resistance to certain topical antimicrobial agents available over-the-counter in the USA (Suzuki* et al.*, 2011). It is likely, therefore, that a continuous selective pressure has been maintaining this element within the USA300 population.

**Fig. 3.**
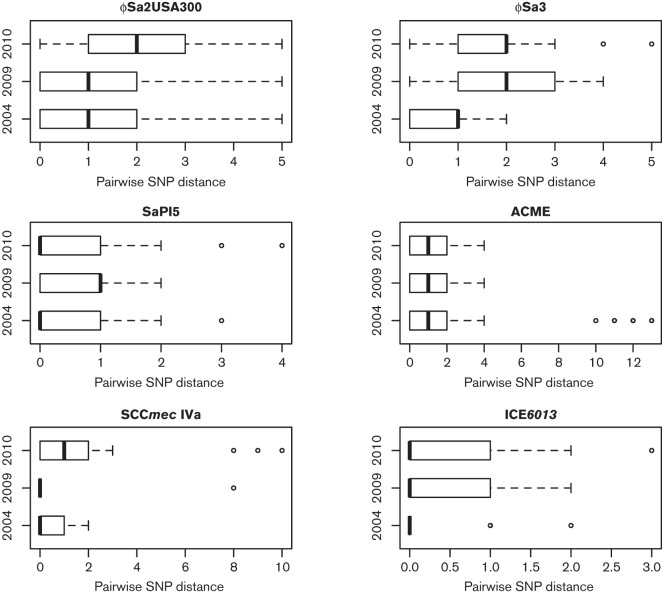
Temporal variation in pairwise SNP distance distributions between chromosomally-inserted MGEs carried by USA300 isolates. Based on isolates derived from healthcare centres selected for temporal analysis (HC1, HC6, HC7, HC10).

We also investigated whether the chromosomally-inserted MGEs carried by USA300 (ϕSa2, ϕSa3, SaPI5, ACME, SCC*mec* IVa and ICE*6013*) diversified over time leading to temporal variation in the distribution of pairwise SNP distances. To discount recombination-mediated variation, alignments of each MGE sequence were first queried for the presence of recombination regions and if detected, the corresponding isolate was removed from the analysis (up to two isolates for ICE*6013, *ϕSa3 and SCC*mec* IVa were removed). We found that the analysed MGEs were well conserved between the isolates, with a median pairwise SNP distance for all elements equal to 1. When comparing the median of pairwise SNP distances for individual MGEs between the 2004, 2009 and 2010 isolates, temporal variation was observed for all except ACME and ICE*6013* elements (Fig. 3). For all, an increase of median pairwise SNP distance by 1 was observed at either or both later sampling time points.

Outside the USA300 clade, it was noteworthy that the temporally distributed USA500-like sub-population differed markedly in its accessory genome from the closely related USA500 clone and could be distinguished based on SCC*mec* type, carriage of *dfrG* insertion element and absence of the ICE*6013*.

### Core genome microevolution of USA300 clade

We investigated further the core genomes of USA300 isolates and found that the population formed a heterogeneous clade with a median pairwise SNP distance of 88 and a range of 0–165 SNPs. We then investigated if USA300 population has become more heterogeneous over time by comparing pairwise SNP distances of isolates collected in 2004 with the panels from 2009 and 2010 (isolates from matched healthcare centres only). We observed an increase in median pairwise SNP distance values between 2004 [62, median absolute deviation (MAD) = 13] and 2009 (103, MAD = 13) as well as 2010 (98, MAD = 15) groups. As previously reported (Kennedy *et al*., 2008), the USA300 isolates demonstrate an elevated ratio of non-synonymous-to-synonymous SNPs (d*N*/d*S*), associated with the recent clonal expansion of this lineage and accumulation of SNPs that have not yet been eliminated by purifying selection (Rocha *et al*., 2006). We observed a d*N*/d*S* median of 0.71 (MAD = 0.19) across USA300 core genomes, in comparison to the median 0.22 (MAD = 0.01) d*N*/d*S* ratio for non-USA300 isolates. In order to determine the d*N*/d*S* ratio of mutations that were acquired independently by the USA300 isolates after each diverged from the last common ancestor, we determined the d*N*/d*S* ratios on terminal branches of the USA300 clade. For clonal or very closely related isolates (up to 2 pairwise SNPs distance) a single representative was selected, and isolates that accumulated less than a total of 10 SNPs since divergence from the last common ancestor, or showed either no synonymous or no non-synonymous mutations were excluded. A total of 139 USA300 isolates were analysed and revealed a median d*N*/d*S* ratio of 2.6 (MAD = 1.1), which corresponded to a previously reported d*N*/d*S* ratio for another USA300 collection (Kennedy *et al*., 2008). Analysis of temporal changes in d*N*/d*S* ratio revealed equally high median values for 2004 (2.33, MAD = 1.23), 2009 (2.5, MAD = 0.4) and 2010 (2.8, MAD = 1.5) altogether indicating that purifying selection has not yet acted upon the USA300 population (Rocha *et al*., 2006).

**Fig. 4.**
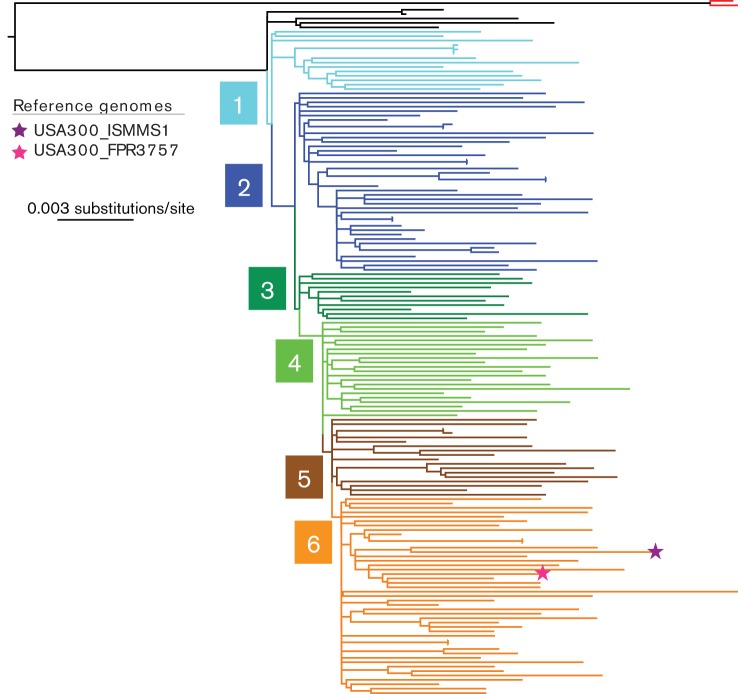
Mid-point rooted phylogenetic tree of *S. aureus* USA300 isolates. Two most closely related non-USA300 isolates were included as an out-group (isolates PFESA2332 and PFESA1492, red branches). Also, two reference genomes were included and corresponding nodes are marked with a star. Branches were highlighted to show consecutive clades of the nested clade structure, and labelled 1 to 6. SNPs that define each labelled clade are described in Table 2.

To interrogate more closely the phylogenetic structure of the USA300 clade, we repeated the phylogeny reconstruction for USA300 isolates only (Fig. 4). Genomic divergence within the USA300 clade, based on branch lengths, was most pronounced on terminal branches and thus was strain-specific. In line with previous studies describing the population structure of USA300 isolates, the generated phylogeny revealed low resolution of inter-strain evolutionary relationships due to short internal branches. We therefore investigated further the clustering patterns of the innermost branches descending immediately from the root of USA300 clade, by focusing on branches that after each evolutionary split gave rise to a majority of the descending population. This revealed a subtle and sequential diversification process involving the formation of nested clades (Fig. 4). Based on the collection analysed in this study, at least six fine-scale divergence events descending from the root of the clade could be identified, each involving acquisition of up to five SNPs (Table 2). Where branches emerged due to multiple SNPs, in each case the mutations were independent in relation to their genomic position and thus did not represent a recombination. Nearly all mutations occurring in protein-coding sequence were non-synonymous (11/12) and involved genes of various functions. Four of the non-synonymous SNPs occurring across three distinct branches were identified in genes associated with antimicrobial resistance. This included a gene encoding a putative drug resistance transporter ErmB/QacA (SAUSA300_2126), a gene encoding an efflux pump (SAUSA300_2489) recently associated with resistance to linoleic and arachidonic acids that are present on the skin and contribute to the low pH of this environment (Alnaseri* et al.*, 2015), and the genes *gyrA* (SAUSA300_006) and *parC/grlA* (SAUSA300_1251) associated with fluoroquinolone resistance. The emergence of fluoroquinolone resistance in the USA300 population was previously described and estimated to have occurred around 1995 (Uhlemann *et al*., 2014). Amongst other genes affected by non-synonymous SNPs was a putative virulence determinant (SAUSA300_1322), which belongs to the *cvfC* operon and contributes to control of haemolysin production and detergent resistance through positive regulation of *thyA* gene (Ikuo* et al.*, 2010).

**Table 2.** Descriptive summary of SNPs identified on branches of early divergence nested clades across the phylogenetic tree of analysed USA300 isolates. The phylogeny is presented in Fig. 4

Clade	FPR3757 Position	SNP Type*	CDS ID	Product	Base change	AA change
1	2300219	NS	SAUSA300_2126	drug resistance transporter, EmrB/QacA subfamily	A -> G	V156A
2	150941	NS	SAUSA300_0132	glycosyl transferase	T -> A	D271E
	714167	I	−	−	A -> G	−
	1453525	NS	SAUSA300_1322	virulence factor C, CvfC3	G -> A	A281V
	1877205	NS	SAUSA300_1701	NAD(FAD)-utilizing dehydrogenase	G -> A	R228H
	1915352	I	−	−	A -> G	−
3	386867	I	−	−	A -> T	−
4	906507	I	**−**	−	C -> A	−
	1693739	NS	SAUSA300_1543	oxygen-independent coproporphyrinogen III oxidase	C -> T	A145T
	1908169	NS	SAUSA300_1725	transaldolase	C -> T	V6I
	2230768	S	SAUSA300_2068	hypothetical protein	T -> C	−
	2690087	NS	SAUSA300_2489	antibiotic transport-associated protein	G -> T	Q795K
5	7282	NS	SAUSA300_0006	DNA gyrase GyrA	C -> T	S84L
	1374405	NS	SAUSA300_1251	DNA topoisomerase ParC/GrlA	C -> A	S80Y
6	837108	NS	SAUSA300_0750	hypothetical protein	T -> G	D17E
	1074815	NS	SAUSA300_0980	putative membrane protein	A -> G	N71D

* SNP type: NS – non-synonymous; I – intergenic; S – synonymou.

To further resolve the observed USA300 tree topology and formation of nested clades, we have reconstructed the phylogeny of the studied USA300 isolates together with 329 genomes of previously sequenced USA300 isolates derived from New York city (Uhlemann *et al*., 2014). The tree topology of this expanded USA300 collection also revealed a nested clade population structure (Fig. S4). The clade -defining mutations corresponded to the polymorphisms described above (Table 2) demonstrating that the early diversification changes identified in this work can be detected across wider USA300 population.

## Conclusions

This study provides an overview of the *S. aureus* CC8 population structure based on whole-genome sequence analysis of clinical isolates derived from nine USA states, collected in 2004, 2009 and 2010. Unsurprisingly, it was dominated by the USA300 clone. The non-USA300 isolates represented a fifth of the CC8 population and included the USA500 clone as well as a closely related USA500-like genotype with a distinct accessory genome composition, which emerged at the later sampling time point. The USA500 clone emerged prior to the appearance and dissemination of the USA300 genotype but has been diminishing in prevalence since (David *et al.*, 2015; Nelson* et al.*, 2015). This trend was also observed in our collection, with the majority of USA500 isolates identified amongst the 2004 samples. Furthermore, the distribution of both USA500 and USA500-like isolates was geographically restricted as most originated from a single site (HC10). This is in contrast to geographically dispersed USA300 isolates. There may also be additional regional differences associated with USA500 that we did not detect owing to the majority of USA500 isolates being detected from just one site. For example, Benson* et al.* (2014) identified that most of the USA500 isolates obtained from patients in New York area harboured the IS256 element located in close proximity to the *rot* gene. In our analysis we identified the presence of this IS element in the genomes of all USA500 isolates and a single USA500-like strain. However, insertion of IS256 next to *rot* was observed for only one of the USA500 strains.

The analysis of temporal variation in the population structure of CC8 isolates indicated a significant drop in the prevalence of USA300 around 2010, in comparison with both 2004 and 2009. However, due to the limitations of our study such as narrow sampling time points, small sample size and lack of isolates representing the Western USA we could not verify if this dip in frequency of USA300 clone represented a broader event or a time trend. A recent review reported that there has been an overall increase in spatio-temporal prevalence of USA300 across the USA between 2000 and 2013 in the context of MRSA epidemiology (Carrel *et al*., 2015). However, regional differences as well as year-on-year fluctuations were also observed, demonstrating a need for a national surveillance program that would allow more accurate monitoring of the epidemiology of clinically significant *S. aureus* clones such as USA300.

Based on this geographically diverse collection of CC8 isolates, we investigated the USA300 clade for evidence of microevolution. The observed limited phylogeographic clustering suggests a general lack of geographical expansion events resulting from emergence of locally adapted clones. This is in agreement with findings that at a local community level, multiple introductions of USA300 occur (Uhlemann *et al*., 2014). Analysis of core genomes revealed that USA300 isolates are becoming more heterogeneous as defined by a temporal increase in pairwise SNP distances, which is underlined by a stable pattern of elevated d*N*/d*S* ratios. However, an analysis of the wider phylogenetic relationships defined by branches descending immediately from the root of the clade revealed the presence of subtle polymorphisms shared across the population. The pattern of accumulative SNP acquisition as defined by the formation of nested clades suggests that the USA300 ancestral isolates continued to diversify shortly after the emergence of the clade but prior to its clonal expansion. Although determining the phenotypic significance of the acquired SNPs is beyond the scope of this work, we speculate that at least some might represent adaptive evolution such as development of antimicrobial resistance.

Our data also suggests temporal changes in the composition of the USA300 accessory genome involving small-scale loss of SCC*mec* and ACME elements and increased frequency of ϕSa5 carriage. These findings are subject to the previously described limitations of this study and thus might not be indicative of a continuous temporal trend of USA300 accessory genome re-shuffle. Still, the collection analysed in this study represents an unbiased sample of *S. aureus* CC8 population derived from diverse clinical sources, providing a unique insight into the broader epidemiology and evolution of USA300 in the context of its parental lineage. This allowed us to capture the apparent instability of SCC*mec* and ACME carriage amongst more recent USA300 isolates. This is significant considering some strategies for identification of USA300 isolates based on defined genetic markers such as carriage of SCC*mec* or ACME (Alam *et al*., 2015; David* et al.*, 2013), which might hinder identification of emerging USA300 variants that have lost these MGEs leading to their under-reporting. Also, taking into consideration the putative role of SCC*mec* and ACME in the successful dissemination of *S. aureus* USA300, it will be important to understand what leads to the loss of these elements. We speculate that the loss of SCC*mec* may have been driven by changes in antimicrobial prescription practices in the USA. It is more challenging to hypothesize what factors could have contributed to the drop in ACME prevalence. However, the observation that absence of SCC*mec* and ACME coincided in majority of isolates, suggests that the elements are frequently lost in a single deletion event. This scenario is supported by previous findings that the CcrAB recombinases of SCC*mec* can mobilise ACME as well as catalyse an excision of the entire ACME– SCC*mec *composite island (Diep* et al.*, 2008). While further work is required to investigate how this might impact on the epidemiology of USA300, the analysis presented here of spatially diverse USA300 isolates gives a new insight into the wider evolutionary changes occurring within this highly successful sub -lineage, and provides ground for further surveillance of this clone.

## Supplementary Data

2896 Supplementary File 1
