# Supplementary material for: Pan-genomic perspective on the evolution of the Staphylococcus aureus USA300 epidemic
Source: Microb Genom. 2016 May 31;2(5):e000058. doi: 10.1099/mgen.0.000058 (PMC5320670; doi:10.1099/mgen.0.000058)

Table S1. Description of 191 CC8 *S. aureus* isolates analysed in this study.

**Public name**	**ENA accession**	**Organism**	**ST**	***spa***	**SCC*mec***	**Clone**	**Year**	**HC ID**	**State**	**Patient type**	**Infection type**
PFESA1490	ERS410907	MSSA	8	t008	-	USA300	2009	HC8	Wisconsin	Outpatient	Other
PFESA1492	ERS410923	MSSA	8	t008	-	-	2009	HC8	Wisconsin	Inpatient	Other
PFESA1606	ERS410796	MSSA	8	t008	-	-	2009	HC4	New York	Inpatient	Invasive
PFESA1614	ERS410765	MSSA	8	t008	-	USA300	2009	HC4	New York	Outpatient	Invasive
PFESA1788	ERS410716	MSSA	8	t008	-	-	2009	HC5	Pennsylvania	Inpatient	Invasive
PFESA1885	ERS410629	MSSA	8	t008	-	USA300	2009	HC3	Missouri	Inpatient	Other
PFESA1975	ERS411176	MSSA	8	t008	-	USA300	2010	HC10	Georgia	Inpatient	Invasive
PFESA1976	ERS411184	MSSA	8	t008	-	USA300	2010	HC10	Georgia	Inpatient	Respiratory
PFESA2034	ERS432065	MSSA	8	t008	-	USA300	2010	HC9	Wisconsin	Outpatient	Other
PFESA2064	ERS365748	MSSA	8	t008	-	USA300	2010	HC7	Texas	Inpatient	Wound
PFESA2070	ERS365796	MSSA	8	t008	-	USA300	2009	HC7	Texas	Inpatient	Wound
PFESA2083	ERS365797	MSSA	8	t008	-	-	2010	HC1	Florida	Inpatient	Invasive
PFESA2084	ERS365805	MSSA	8	t008	-	USA300	2010	HC1	Florida	Inpatient	Other
PFESA2091	ERS365766	MSSA	8	t008	-	USA300	2010	HC1	Florida	Inpatient	Other
PFESA2161	ERS365815	MSSA	8	t008	-	USA300	2010	HC2	Michigan	Inpatient	Respiratory
PFESA2175	ERS410378	MSSA	8	t008	-	-	2010	HC2	Michigan	Outpatient	Respiratory
PFESA2178	ERS410402	MSSA	8	t008	-	USA300	2009	HC11	Florida	Inpatient	Other
PFESA2180	ERS410418	MSSA	8	t008	-	USA300	2009	HC11	Florida	Inpatient	Wound
PFESA2184	ERS410450	MSSA	8	t008	-	USA300	2009	HC11	Florida	Inpatient	Other
PFESA2196	ERS410451	MSSA	8	t008	-	-	2010	HC12	Pennsylvania	Inpatient	Wound
PFESA2274	ERS365784	MSSA	8	t008	-	USA300	2010	HC6	Tennessee	Inpatient	Invasive
PFESA2298	ERS365754	MSSA	8	t008	-	USA300	2010	HC2	Michigan	Inpatient	Wound
PFESA2303	ERS365794	MSSA	8	t008	-	-	2010	HC2	Michigan	Inpatient	Other
PFESA2332	ERS410342	MSSA	8	t008	-	-	2010	HC8	Wisconsin	Outpatient	Wound
PFESA2413	ERS365826	MSSA	8	t008	-	USA300	2010	HC7	Texas	Inpatient	Respiratory

Table S1. Description of 191 CC8 *S. aureus* isolates analysed in this study.

**Public name**	**ENA accession**	**Organism**	**ST**	***spa***	**SCC*mec***	**Clone**	**Year**	**HC ID**	**State**	**Patient type**	**Infection type**
PFESA2417	ERS411049	MSSA	8	t008	-	USA300	2010	HC7	Texas	Inpatient	Respiratory
PFESA2432	ERS410215	MSSA	8	t008	-	-	2010	HC5	Pennsylvania	Inpatient	Invasive
PFESA2449	ERS432202	MSSA	8	t008	-	USA300	2010	HC3	Missouri	Inpatient	Wound
PFESA2453	ERS432139	MSSA	8	t008	-	USA300	2010	HC9	Wisconsin	Outpatient	Wound
PFESA2460	ERS432195	MSSA	8	t008	-	USA300	2010	HC9	Wisconsin	Inpatient	Other
PFESA1306	ERS411147	MRSA	8	t008	IVa	USA300	2004	HC7	Texas	Inpatient	Wound
PFESA1308	ERS411163	MRSA	8	t008	IVa	USA300	2004	HC7	Texas	Inpatient	Invasive
PFESA1309	ERS411171	MRSA	8	t008	IVa	USA300	2004	HC7	Texas	Inpatient	Wound
PFESA1311	ERS411179	MRSA	8	t008	IVa	USA300	2004	HC7	Texas	Inpatient	Wound
PFESA1312	ERS411187	MRSA	8	t008	IVa	USA300	2004	HC7	Texas	Inpatient	Wound
PFESA1317	ERS411132	MRSA	8	t008	IVa	USA300	2004	HC7	Texas	Inpatient	Invasive
PFESA1322	ERS411172	MRSA	8	t008	IVa	USA300	2004	HC7	Texas	Inpatient	Wound
PFESA1326	ERS411112	MRSA	8	t008	IVa	USA300	2004	HC1	Florida	Outpatient	Wound
PFESA1328	ERS411204	MRSA	8	t008	IVa	USA300	2004	HC1	Florida	Inpatient	Wound
PFESA1329	ERS410929	MRSA	8	t008	IVa	USA300	2004	HC1	Florida	Outpatient	Wound
PFESA1333	ERS411133	MRSA	8	t008	IVa	USA300	2004	HC1	Florida	Inpatient	Invasive
PFESA1335	ERS411213	MRSA	8	t008	IVa	USA300	2004	HC1	Florida	Inpatient	Respiratory
PFESA1339	ERS411205	MRSA	8	t008	IVa	USA300	2004	HC1	Florida	Inpatient	Wound
PFESA1341	ERS410977	MRSA	8	t008	IVa	USA300	2004	HC1	Florida	Outpatient	Other
PFESA1342	ERS410985	MRSA	8	t008	IVa	USA300	2004	HC1	Florida	Outpatient	Wound
PFESA1345	ERS411009	MRSA	8	t008	IVa	USA300	2004	HC1	Florida	Outpatient	Other
PFESA1347	ERS411017	MRSA	8	t008	IVa	USA300	2004	HC1	Florida	Outpatient	Wound
PFESA1350	ERS411173	MRSA	8	t008	IVa	USA300	2004	HC10	Georgia	Inpatient	Wound
PFESA1352	ERS410946	MRSA	8	t008	IVa	USA300	2004	HC10	Georgia	Outpatient	Wound
PFESA1353	ERS410954	MRSA	8	t008	IVa	USA300	2004	HC10	Georgia	Outpatient	Wound

Table S1. Description of 191 CC8 *S. aureus* isolates analysed in this study.

Public name	ENA accession	Organism	ST	*spa*	SCC*mec*	Clone	Year	HC ID	State	Patient type	Infection type
PFESA1354	ERS410962	MRSA	8	t008	IVa	USA300	2004	HC10	Georgia	Outpatient	Wound
PFESA1355	ERS410970	MRSA	8	t008	IVa	USA300	2004	HC10	Georgia	Outpatient	Other
PFESA1357	ERS410986	MRSA	8	t008	IVa	USA300	2004	HC10	Georgia	Outpatient	Other
PFESA1358	ERS410994	MRSA	8	t008	IVa	USA300	2004	HC10	Georgia	Outpatient	Other
PFESA1359	ERS411002	MRSA	8	t008	IVa	USA300	2004	HC10	Georgia	Outpatient	Other
PFESA1360	ERS411010	MRSA	8	t008	IVa	USA300	2004	HC10	Georgia	Outpatient	Other
PFESA1362	ERS411018	MRSA	8	t008	IVa	USA300	2004	HC10	Georgia	Outpatient	Other
PFESA1364	ERS410939	MRSA	8	t008	IVa	USA300	2004	HC10	Georgia	Outpatient	Other
PFESA1365	ERS411157	MRSA	8	t008	IVa	USA300	2004	HC10	Georgia	Inpatient	Other
PFESA1366	ERS411149	MRSA	8	t008	IVa	USA300	2004	HC10	Georgia	Inpatient	Other
PFESA1368	ERS411141	MRSA	8	t008	IVa	USA300	2004	HC10	Georgia	Inpatient	Wound
PFESA1369	ERS410955	MRSA	8	t008	IVa	USA300	2004	HC10	Georgia	Outpatient	Other
PFESA1371	ERS411126	MRSA	8	t008	IVa	USA300	2004	HC10	Georgia	Inpatient	Other
PFESA1391	ERS411150	MRSA	8	t008	IVa	USA300	2004	HC6	Tennessee	Inpatient	Other
PFESA1398	ERS411020	MRSA	8	t008	IVa	USA300	2004	HC6	Tennessee	Outpatient	Wound
PFESA1399	ERS410933	MRSA	8	t008	IVa	USA300	2004	HC6	Tennessee	Outpatient	Wound
PFESA1404	ERS410949	MRSA	8	t008	IVa	USA300	2004	HC6	Tennessee	Outpatient	Other
PFESA1410	ERS411167	MRSA	8	t008	IVa	USA300	2004	HC6	Tennessee	Inpatient	Other
PFESA1476	ERS410890	MRSA	8	t008	IVa	USA300	2010	HC8	Wisconsin	Inpatient	Wound
PFESA1477	ERS410898	MRSA	8	t008	IVa	USA300	2010	HC8	Wisconsin	Outpatient	Other
PFESA1485	ERS410867	MRSA	8	t008	IVa	USA300	2009	HC8	Wisconsin	Inpatient	Other
PFESA1489	ERS410899	MRSA	8	t008	IVa	USA300	2009	HC8	Wisconsin	Outpatient	Wound
PFESA1491	ERS410915	MRSA	8	t008	IVa	USA300	2009	HC8	Wisconsin	Outpatient	Wound
PFESA1497	ERS410868	MRSA	8	t008	IVa	USA300	2009	HC8	Wisconsin	Inpatient	Other
PFESA1498	ERS410876	MRSA	8	t008	IVa	USA300	2009	HC8	Wisconsin	Inpatient	Other

Table S1. Description of 191 CC8 *S. aureus* isolates analysed in this study.

**Public name**	**ENA accession**	**Organism**	**ST**	***spa***	**SCC*mec***	**Clone**	**Year**	**HC ID**	**State**	**Patient type**	**Infection type**
PFESA1532	ERS410863	MRSA	8	t008	IVa	USA300	2009	HC12	Pennsylvania	Inpatient	Other
PFESA1537	ERS410903	MRSA	8	t008	IVa	USA300	2009	HC12	Pennsylvania	unknown	Wound
PFESA1538	ERS410911	MRSA	8	t008	IVa	USA300	2009	HC12	Pennsylvania	Inpatient	Respiratory
PFESA1541	ERS410840	MRSA	8	t008	IVa	USA300	2009	HC12	Pennsylvania	Inpatient	Other
PFESA1545	ERS410872	MRSA	8	t008	IVa	USA300	2009	HC12	Pennsylvania	Inpatient	Wound
PFESA1547	ERS410888	MRSA	8	t008	IVa	USA300	2009	HC12	Pennsylvania	Inpatient	Respiratory
PFESA1579	ERS410747	MRSA	8	t008	IVa	USA300	2009	HC6	Tennessee	Outpatient	Wound
PFESA1580	ERS410755	MRSA	8	t008	IVa	USA300	2009	HC6	Tennessee	Outpatient	Wound
PFESA1583	ERS410771	MRSA	8	t008	IVa	USA300	2009	HC6	Tennessee	Outpatient	Other
PFESA1584	ERS410779	MRSA	8	t008	IVa	USA300	2009	HC6	Tennessee	Outpatient	Wound
PFESA1585	ERS411199	MRSA	8	t008	IVa	USA300	2009	HC6	Tennessee	Inpatient	Wound
PFESA1586	ERS410787	MRSA	8	t008	IVa	USA300	2009	HC6	Tennessee	Outpatient	Other
PFESA1589	ERS410811	MRSA	8	t008	IVa	USA300	2009	HC6	Tennessee	Outpatient	Wound
PFESA1590	ERS411207	MRSA	8	t008	IVa	USA300	2009	HC6	Tennessee	Inpatient	Other
PFESA1591	ERS410819	MRSA	8	t008	IVa	USA300	2009	HC6	Tennessee	Outpatient	Other
PFESA1593	ERS410740	MRSA	8	t008	IVa	USA300	2009	HC6	Tennessee	Outpatient	Other
PFESA1594	ERS410748	MRSA	8	t008	IVa	USA300	2009	HC6	Tennessee	Outpatient	Other
PFESA1618	ERS410797	MRSA	8	t008	IVa	USA300	2009	HC4	New York	Outpatient	Wound
PFESA1785	ERS410692	MRSA	8	t008	IVa	USA300	2009	HC5	Pennsylvania	Inpatient	Respiratory
PFESA1787	ERS410708	MRSA	8	t008	IVa	USA300	2009	HC5	Pennsylvania	Inpatient	Respiratory
PFESA1791	ERS410645	MRSA	8	t008	IVa	USA300	2009	HC5	Pennsylvania	Inpatient	Respiratory
PFESA1880	ERS410589	MRSA	8	t008	IVa	USA300	2010	HC3	Missouri	Inpatient	Other
PFESA1882	ERS410605	MRSA	8	t008	IVa	USA300	2009	HC3	Missouri	Inpatient	Other
PFESA1883	ERS410613	MRSA	8	t008	IVa	USA300	2010	HC3	Missouri	Inpatient	Wound
PFESA1888	ERS410558	MRSA	8	t008	IVa	USA300	2010	HC3	Missouri	Inpatient	Other

Table S1. Description of 191 CC8 *S. aureus* isolates analysed in this study.

Public name	ENA accession	Organism	ST	*spa*	SCC*mec*	Clone	Year	HC ID	State	Patient type	Infection type
PFESA1889	ERS410566	MRSA	8	t008	IVa	USA300	2010	HC3	Missouri	Inpatient	Other
PFESA1977	ERS432053	MRSA	8	t008	IVa	USA300	2010	HC10	Georgia	Outpatient	Other
PFESA1978	ERS432061	MRSA	8	t008	IVa	USA300	2010	HC10	Georgia	Outpatient	Other
PFESA2055	ERS365779	MRSA	8	t008	IVa	USA300	2009	HC7	Texas	Inpatient	Invasive
PFESA2057	ERS365795	MRSA	8	t008	IVa	USA300	2009	HC7	Texas	Inpatient	Invasive
PFESA2059	ERS365811	MRSA	8	t008	IVa	USA300	2009	HC7	Texas	Inpatient	Other
PFESA2062	ERS365827	MRSA	8	t008	IVa	USA300	2010	HC7	Texas	Inpatient	Wound
PFESA2063	ERS365740	MRSA	8	t008	IVa	USA300	2009	HC7	Texas	Inpatient	Wound
PFESA2067	ERS365772	MSSA	8	t008	IVa	USA300	2009	HC7	Texas	Inpatient	Other
PFESA2074	ERS365828	MRSA	8	t008	IVa	USA300	2009	HC7	Texas	Inpatient	Wound
PFESA2075	ERS410365	MRSA	8	t008	IVa	USA300	2010	HC7	Texas	Outpatient	Invasive
PFESA2077	ERS365749	MRSA	8	t008	IVa	USA300	2010	HC1	Florida	Inpatient	Wound
PFESA2090	ERS365758	MRSA	8	t008	IVa	USA300	2010	HC1	Florida	Inpatient	Wound
PFESA2098	ERS365822	MRSA	8	t008	IVa	USA300	2010	HC1	Florida	Inpatient	Wound
PFESA2103	ERS365767	MRSA	8	t008	IVa	USA300	2010	HC1	Florida	Inpatient	Respiratory
PFESA2104	ERS365775	MRSA	8	t008	IVa	USA300	2010	HC1	Florida	Inpatient	Respiratory
PFESA2153	ERS410377	MRSA	8	t008	IVa	USA300	2010	HC2	Michigan	Outpatient	Other
PFESA2157	ERS365791	MRSA	8	t008	IVa	USA300	2010	HC2	Michigan	Inpatient	Wound
PFESA2158	ERS410401	MRSA	8	t008	IVa	USA300	2010	HC2	Michigan	Outpatient	Other
PFESA2163	ERS410409	MRSA	8	t008	IVa	USA300	2010	HC2	Michigan	Outpatient	Wound
PFESA2164	ERS365831	MRSA	8	t008	IVa	USA300	2010	HC2	Michigan	Inpatient	Wound
PFESA2166	ERS410417	MRSA	8	t008	IVa	USA300	2010	HC2	Michigan	Outpatient	Other
PFESA2169	ERS410441	MRSA	8	t008	IVa	USA300	2010	HC2	Michigan	Outpatient	Wound
PFESA2188	ERS410387	MRSA	8	t008	IVa	USA300	2009	HC11	Florida	Inpatient	Respiratory
PFESA2192	ERS410419	MRSA	8	t008	IVa	USA300	2009	HC12	Pennsylvania	Outpatient	Wound

Table S1. Description of 191 CC8 *S. aureus* isolates analysed in this study. For *spa* and SCC*mec* columns NT refers to non-typeable.

**Public name**	**ENA accession**	**Organism**	**ST**	***spa***	**SCC*mec***	**Clone**	**Year**	**HC ID**	**State**	**Patient type**	**Infection type**
PFESA2193	ERS410427	MRSA	8	t008	IVa	USA300	2009	HC12	Pennsylvania	Inpatient	Wound
PFESA2272	ERS410276	MRSA	8	t008	IVa	USA300	2010	HC6	Tennessee	Outpatient	Wound
PFESA2273	ERS410284	MRSA	8	t008	IVa	USA300	2010	HC6	Tennessee	Outpatient	Other
PFESA2282	ERS365745	MRSA	8	t008	IVa	USA300	2010	HC6	Tennessee	Inpatient	Respiratory
PFESA2283	ERS410300	MRSA	8	t008	IVa	USA300	2010	HC6	Tennessee	Outpatient	Other
PFESA2285	ERS410308	MRSA	8	t008	IVa	USA300	2010	HC6	Tennessee	Outpatient	Other
PFESA2329	ERS410318	MRSA	8	t008	IVa	USA300	2010	HC8	Wisconsin	Outpatient	Other
PFESA2431	ERS410207	MRSA	8	t008	IVa	USA300	2010	HC5	Pennsylvania	Inpatient	Respiratory
PFESA2435	ERS410239	MRSA	8	t008	IVa	USA300	2010	HC5	Pennsylvania	Inpatient	Respiratory
PFESA2293	ERS365809	MRSA	8	t008	IVg	-	2010	HC2	Michigan	Inpatient	Invasive
PFESA2056	ERS365787	MRSA	8	t008	NT	USA300	2009	HC7	Texas	Inpatient	Respiratory
PFESA1884	ERS410621	MSSA	8	t024	-	-	2009	HC3	Missouri	Inpatient	Other
PFESA2176	ERS410386	MSSA	8	t024	-	USA300	2009	HC11	Florida	Inpatient	Wound
PFESA1304	ERS411131	MRSA	8	t024	IVa	USA300	2004	HC7	Texas	Inpatient	Invasive
PFESA1337	ERS410961	MRSA	8	t024	IVa	USA300	2004	HC1	Florida	Outpatient	Other
PFESA1346	ERS411189	MRSA	8	t024	IVa	USA300	2004	HC1	Florida	Inpatient	Wound
PFESA1388	ERS410996	MRSA	8	t024	IVa	USA300	2004	HC6	Tennessee	Outpatient	Invasive
PFESA1598	ERS410756	MRSA	8	t024	IVa	USA300	2009	HC6	Tennessee	Outpatient	Other
PFESA2156	ERS410393	MRSA	8	t024	IVa	USA300	2010	HC2	Michigan	Outpatient	Other
PFESA2469	ERS432172	MRSA	8	t024	IVa	USA300	2010	HC9	Wisconsin	Inpatient	Wound
PFESA1795	ERS410677	MSSA	8	t064	-	-	2009	HC5	Pennsylvania	Inpatient	Respiratory
PFESA2439	ERS432122	MSSA	8	t064	-	-	2010	HC5	Pennsylvania	Inpatient	Respiratory
PFESA1318	ERS411140	MRSA	8	t064	IVd	USA500	2004	HC7	Texas	Inpatient	Wound
PFESA1351	ERS410938	MRSA	8	t064	IVd	USA500	2004	HC10	Georgia	Outpatient	Wound
PFESA1361	ERS411165	MRSA	8	t064	IVd	USA500	2004	HC10	Georgia	Inpatient	Invasive

Table S1. Description of 191 CC8 *S. aureus* isolates analysed in this study.

Public name	ENA accession	Organism	ST	*spa*	SCC*mec*	Clone	Year	HC ID	State	Patient type	Infection type
PFESA1987	ERS365739	MRSA	8	t064	IVd	USA500	2010	HC10	Georgia	Inpatient	Invasive
PFESA1974	ERS411168	MRSA	8	t064	IVg	USA500-like	2009	HC10	Georgia	Inpatient	Respiratory
PFESA1980	ERS432077	MRSA	8	t064	IVg	USA500-like	2010	HC10	Georgia	Outpatient	Other
PFESA1984	ERS411200	MRSA	8	t064	IVg	USA500-like	2010	HC10	Georgia	Inpatient	Respiratory
PFESA1985	ERS411208	MRSA	8	t064	IVg	USA500-like	2010	HC10	Georgia	Inpatient	Invasive
PFESA2086	ERS365821	MRSA	8	t064	IVg	USA500-like	2010	HC1	Florida	Inpatient	Wound
PFESA2199	ERS410380	MRSA	8	t064	IVg	USA500-like	2010	HC12	Pennsylvania	Inpatient	Respiratory
PFESA1327	ERS411120	MRSA	8	t068	IVa	USA300	2004	HC1	Florida	Outpatient	Wound
PFESA1367	ERS410947	MRSA	8	t121	IVa	USA300	2004	HC10	Georgia	Outpatient	Other
PFESA2281	ERS234133	MRSA	8	t121	IVa	USA300	2010	HC6	Tennessee	Inpatient	Respiratory
PFESA2277	ERS365808	MSSA	8	t1348	-	-	2010	HC6	Tennessee	Inpatient	Wound
PFESA2087	ERS365829	MRSA	8	t1578	IVa	USA300	2010	HC1	Florida	Inpatient	Wound
PFESA1989	ERS365755	MSSA	8	t1705	-	-	2010	HC10	Georgia	Inpatient	Other
PFESA2094	ERS365790	MSSA	8	t1882	-	-	2010	HC1	Florida	Inpatient	Respiratory
PFESA2170	ERS410449	MRSA	8	t211	IVa	USA300	2010	HC2	Michigan	Outpatient	Other
PFESA2187	ERS410379	MSSA	8	t2179	-	USA300	2009	HC11	Florida	Inpatient	Wound
PFESA1390	ERS411142	MSSA	8	t2558	-	-	2004	HC6	Tennessee	Inpatient	Other
PFESA2331	ERS410334	MSSA	8	t3240	-	-	2010	HC8	Wisconsin	Outpatient	Wound
PFESA1478	ERS410906	MSSA	8	t334	-	-	2009	HC8	Wisconsin	Inpatient	Respiratory
PFESA2280	ERS365824	MSSA	8	t334	-	-	2010	HC6	Tennessee	Inpatient	Respiratory
PFESA1370	ERS410963	MRSA	8	t4166	IVa	USA300	2004	HC10	Georgia	Outpatient	Other
PFESA1890	ERS410574	MRSA	8	t622	IVa	USA300	2010	HC3	Missouri	Inpatient	Wound
PFESA1982	ERS432085	MRSA	8	t622	IVa	USA300	2010	HC10	Georgia	Outpatient	Other
PFESA1981	ERS411192	MRSA	8	t681	IVa	USA300	2010	HC10	Georgia	Inpatient	Respiratory
PFESA1990	ERS365763	MRSA	8	t681	IVa	USA300	2010	HC10	Georgia	Inpatient	Other

Table S1. Description of 191 CC8 *S. aureus* isolates analysed in this study. For *spa* and SCC*mec* columns NT refers to non-typeable.

**Public name**	**ENA accession**	**Organism**	**ST**	***spa***	**SCC*mec***	**Clone**	**Year**	**HC ID**	**State**	**Patient type**	**Infection type**
PFESA2763	ERS409806	MRSA	8	t681	IVa	USA300	2010	HC2	Michigan	unknown	Other
PFESA2742	ERS409790	MSSA	8	t8286	-	-	2010	HC2	Michigan	unknown	Other
PFESA1344	ERS411001	MRSA	8	NT	IVa	USA300	2004	HC1	Florida	Outpatient	Wound
PFESA1988	ERS365747	MRSA	8	NT	IVa	USA300	2009	HC10	Georgia	Inpatient	Invasive
PFESA2330	ERS410326	MSSA	976	t008	-	-	2010	HC8	Wisconsin	Outpatient	Other
PFESA2279	ERS365816	MSSA	1150	NT	-	-	2010	HC6	Tennessee	Inpatient	Wound
PFESA1548	ERS410896	MRSA	2253	t008	IVg	-	2010	HC12	Pennsylvania	Inpatient	Invasive
PFESA2189	ERS410395	MRSA	2253	t008	IVg	-	2010	HC12	Pennsylvania	Inpatient	Wound
PFESA2203	ERS410412	MRSA	2253	t008	IVg	-	2010	HC12	Pennsylvania	Inpatient	Wound
PFESA2073	ERS365820	MRSA	2319	t008	IVa	USA300	2009	HC7	Texas	Inpatient	Wound
PFESA1617	ERS410789	MRSA	3000	t008	IVa	USA300	2010	HC4	New York	Inpatient	Wound
PFESA2473	ERS432204	MSSA	3001	t121	-	-	2010	HC9	Wisconsin	Inpatient	Wound
PFESA2278	ERS410292	MRSA	3007	t008	IVa	USA300	2010	HC6	Tennessee	Outpatient	Wound
PFESA1983	ERS432093	MRSA	3008	t008	IVa	USA300	2010	HC10	Georgia	Outpatient	Other
PFESA2054	ERS409812	MRSA	3010	t008	IVa	USA300	2009	HC7	Texas	Outpatient	Wound
PFESA1542	ERS410848	MRSA	3015	t008	IVa	USA300	2009	HC12	Pennsylvania	Inpatient	Other

Figure S1. Midpoint-rooted phylogenetic tree of analysed *S. aureus* CC8 isolates with distribution of MGE-associated virulence and resistance genes. Four reference genomes were included (corresponding nodes marked with a star). Branches of USA300, USA500 and USA500-like clades have been highlighted. The tree has been annotated to show the variable distribution of virulence genes associated with prophages ϕSa2 (*pvl*) and ϕSa3 (*sak, scn, chp, seK, seQ, seA*) as well as the distribution of antimicrobial resistance genes associated with plasmids pUSA300HOUMR-like (*blaZ, smrA, mphBM, aphA-3, bcrA, sat*) and pUSA03-like (*ileS, aadD*).

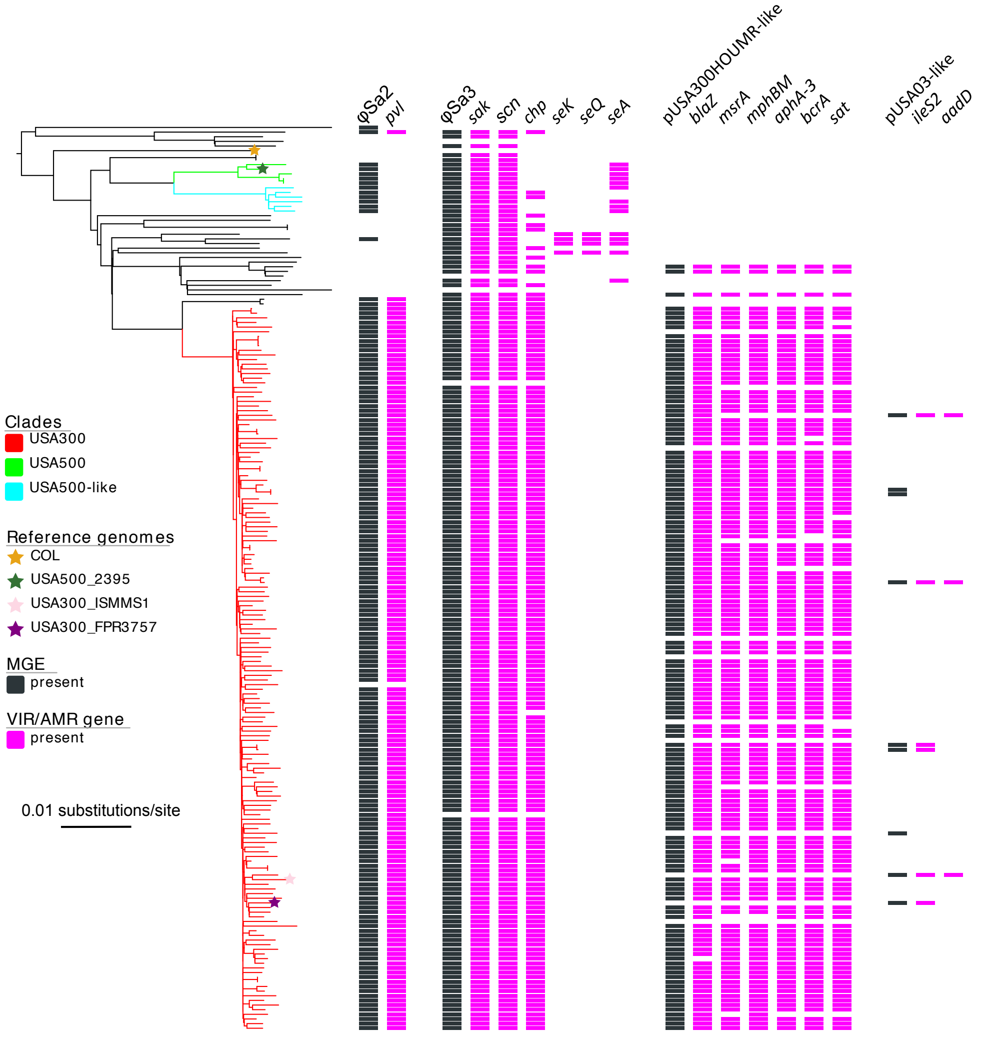

Figure S2. Comparative alignment of recombinant variants of SCC*mec* and ACME elements identified in USA300 isolates. (a) Alignment of SCC*mec*-ACME hybrid identified in isolate PFESA2056 to SCC*mec*IVa-ACME region of S. *aureus* USA300_FPR3757 strain. (b) Alignment of SCC*mec* hybrid identified in isolate PFESA2090 to sequence of SCC*mec* IVa from USA300_FPR3757 and SCC*mec* V (5&5C2) from S. *aureus* JCSC6944 strains. (c) Alignment of region containing remnant SCC and ACME element identified in isolate PFESA2064 to SCC*mec*IVa-ACME region of USA300_FPR3757 strain and SCCpbp4 from S. *epidermidis* ATCC 12228. Alignment figures were constructed using Easyfig software v 2.1

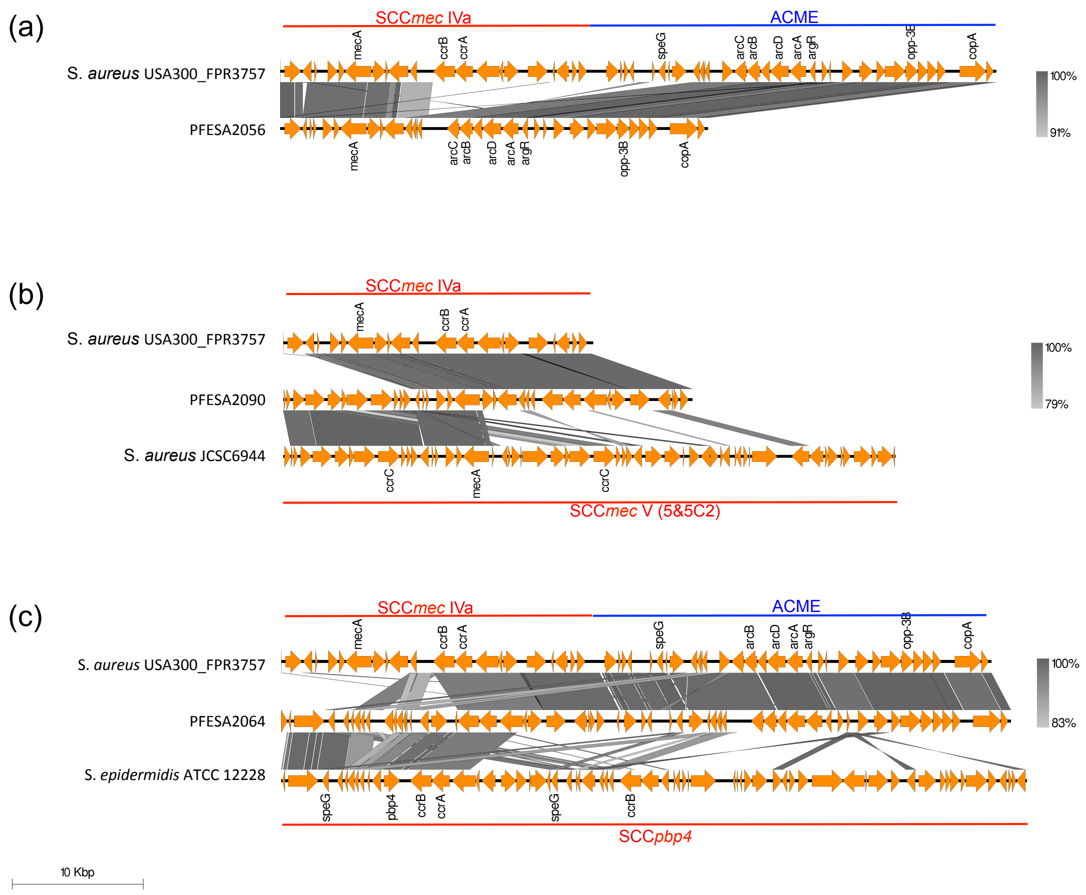

Figure S3. **Midpoint-rooted phylogenetic tree of analysed *S. aureus* CC8 isolates showing distribution of the ICE6013 mobile genetic element**. Four reference genomes were included (corresponding nodes marked with a star). Branches of USA300, USA500 and USA500-like clades have been highlighted. Tree has been annotated to show correlation between phylogenetic distribution and, starting from the innermost annotation circle, ICE6013 carriage, ICE6013 insertion type (intergenic or intragenic) and ICE6013 insertion site. For the latter, name of the coding sequence from USA300_FPR3757 reference genome was used, for intergenic location sites names of the flanking coding sequences are provided.

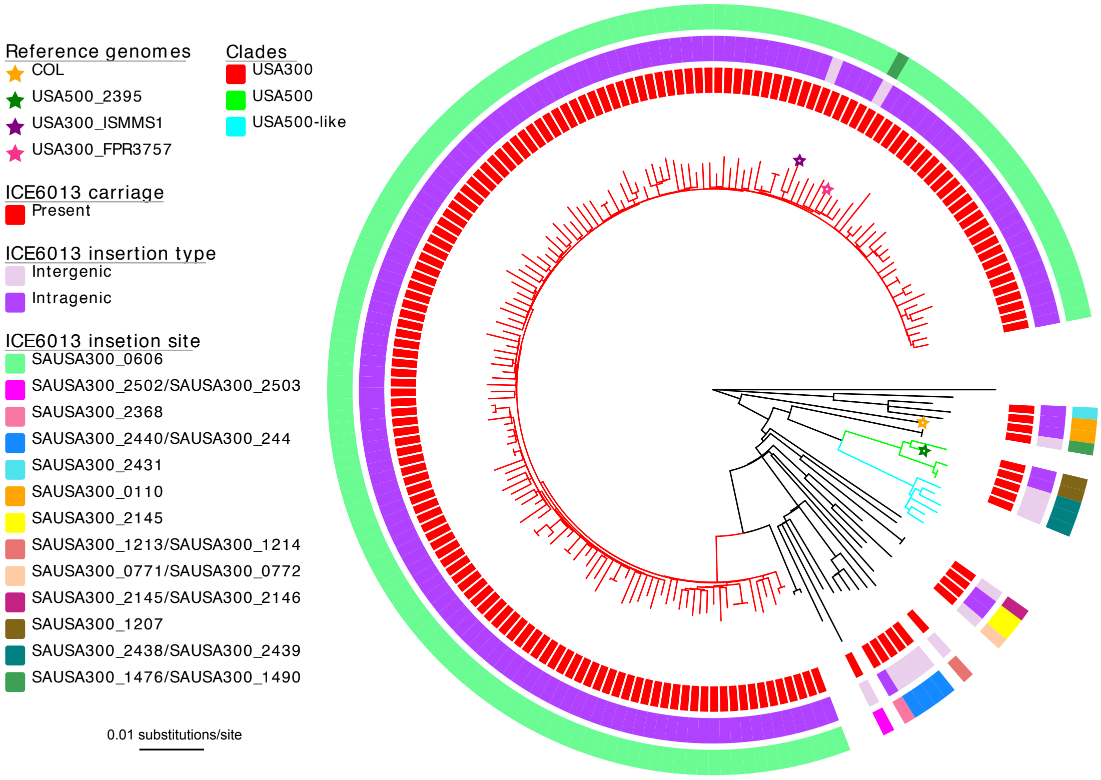

Figure S4. Mid-point rooted phylogenetic tree of 483 *S. aureus* USA300 isolates. The phylogeny consists of 154 USA300 isolates analysed in this study together with 329 previously published USA300 genomes derived from New York City (Uhlemann, Dordel et al. 2014). Two most closely related non-USA300 isolates were included as an out-group (isolates PFESA2332 and PFESA1492, branches coloured in red). Branches were coloured to highlight the phylogeny's nested clade structure. Clades were labelled from 1 to 6, to illustrate a parallel population structure between USA300 isolates analysed in this study and this expanded collection. SNPs that define each labelled clade correspond to previously described polymorphisms (Table 1), except for clade 5 where mutation at site corresponding to 1374405 position in USA300_FPR3757, was detected as C ➔ T transition (5a) followed by T ➔ A transversion (5b), in contrast to previously described single step C ➔ A transversion. Two reference genomes were included and corresponding nodes are marked with a star.

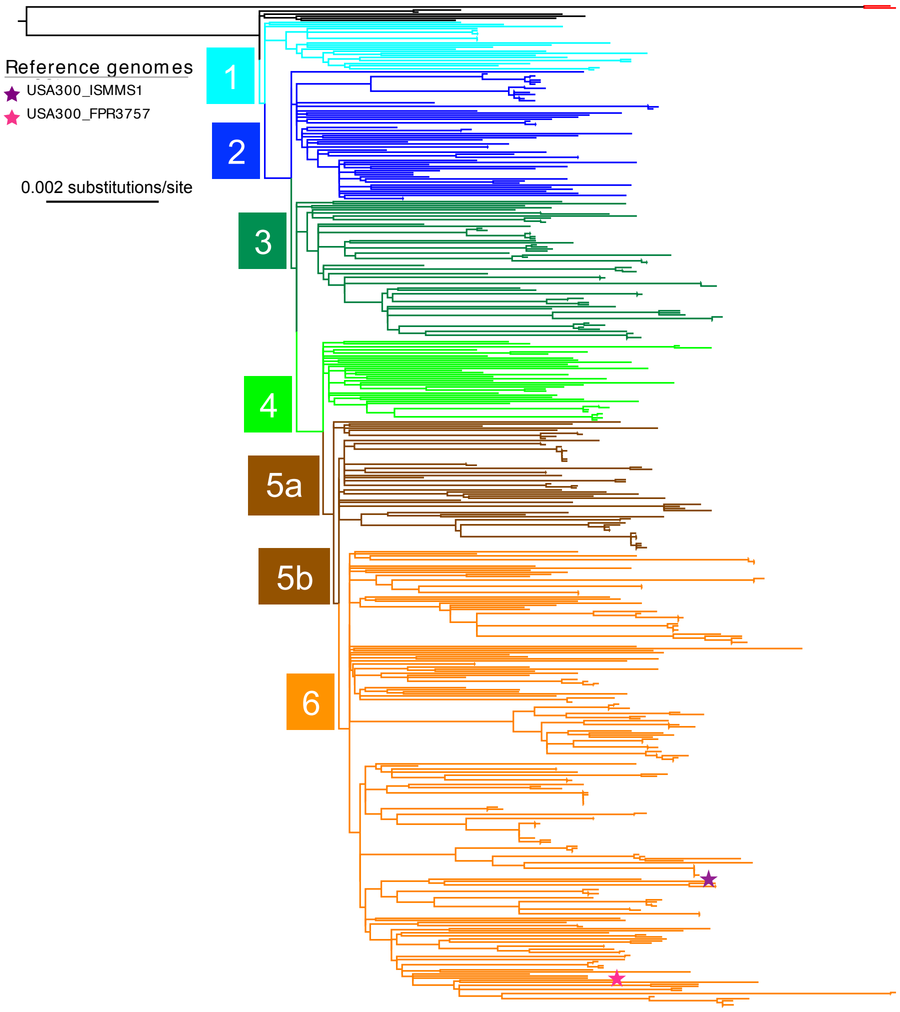

Supplement: Supplementary file 1 [file mgen-02-58-s001.pdf]
